# Role of *β*2 Integrins in the Binding of Thymocytes to Rat Thymic Macrophages

**DOI:** 10.1155/1994/95687

**Published:** 1994

**Authors:** Miodrag Čolić, Vesna Ilić, Takuya Tamatani, Masayuki Miyasaka, Miloš D. Pavlović

**Affiliations:** 1 lnstitute of Medical Research Military Medical Academy Crnotravska 17 Belgrade Serbia and Montenegro; 2 Department of Immunology Tokyo Metropolitan Institute of Medical Science 3-18-22 Hon-Komagome, Bun Kyo Tokyo 113 Japan

**Keywords:** Thymic macrophages, thymocytes, adhesion molecules, cytokines, rosettes

## Abstract

A role of β2 integrins and one of their ligands, ICAM-1, in thymic macrophage (TMF)/thymocyte interactions was studied. TMF were isolated as adherent cells from 4-day old culture of thymic-cell suspensions either from normal or hydrocortisone-treated rats. Adherent cells were 94-98% positive with ED1 (a pan-macrophage marker). The majority of them (75-95%) expressed the CD11b and CD18 molecules, and 60-70% expressed CD54 (ICAM-1). A low proportion of TMF (10-20%) expressed CDlla (LFA-1). The expression of all these antigens was upregulated by IFN-α and TNF-α. The effect of these mAbs on TMF/thymocyte binding was studied using a simple rosette assay by incubating unstimulated or IFN-γ or TNF-α stimulated TMF, grown on microscopic slides with resting or ConA +IL-2 activated thymocytes. It was found that LFA-1/CD18 and ICAM-1 play a significant role in the TMF/thymocyte adhesion. In addition, a LFA-l-dependent/ICAM- 1-independent adhesion pathway was observed, suggesting that LFA-1 might use another ligand. The inhibitory effect of anti-CD18 mAb (WT-3) was higher than the effect of anti-LFA-1 mAb (WT-1) and was a consequence of blocking the CD18 chain both on thymocytes and TMF. No significant difference in the expression and function of adhesion molecules was found between TMF obtained from normal or hydrocortisone-treated rats. The involvement of CD1 1b in these processes was of lesser importance than the role of the CD11a molecule. By using mAbs to different epitopes of the CD11b molecule, such as OX-42 (anti-CD11b/CD11c), ED7, and ED8 (anti-CD11b), it was found that they were either slightly or moderately inhibitory under certain experimental conditions or did not significantly modulate TMF/thymocyte binding. Oχ-42 was slightly stimulatory in some experiments. Cumulatively, these results show that 2 integrins play a significant role in TMF/thymocyte interactions and probably contribute to T-cell development in vivo.


*Developmental Immunology*, 1994, Vol 4, pp. 65–77
Reprints available directly from the publisher
Photocopying permitted by license only

© 1994 Harwood Academic Publishers GmbH
Printed in Singapore

### Role of β2 Integrins in the Binding of Thymocytes to Rat Thymic Macrophages

MIODRAG ČOLIĆ,[†*] VESNA ILIĆ,[†] TAKUYA TAMATANI,[‡] MASAYUKI MIYASAKA[‡] and MILOŠ D. PAVLOVIĆ[†]

[†]*Institute of Medical Research, Military Medical Academy, Crnotravska 17, Belgrade, Yugoslavia*
[‡]*Department of Immunology, Tokyo Metropolitan Institute of Medical Science, 3-18-22, Hon-Komagome, Bun Kyo, Tokyo 113, Japan*

A role of β2 integrins and one of their ligands, ICAM-1, in thymic macrophage (TMF)/thymocyte interactions was studied. TMF were isolated as adherent cells from 4-day old culture of thymic-cell suspensions either from normal or hydrocortisone-treated rats. Adherent cells were 94–98% positive with ED1 (a pan-macrophage marker). The majority of them (75–95%) expressed the CD11b and CD18 molecules, and 60–70% expressed CD54 (ICAM-1). A low proportion of TMF (10–20%) expressed CD11a (LFA-1). The expression of all these antigens was upregulated by IFN-γ and TNF-α. The effect of these mAbs on TMF/thymocyte binding was studied using a simple rosette assay by incubating unstimulated or IFN-γ or TNF-α stimulated TMF, grown on microscopic slides with resting or ConA +IL-2 activated thymocytes. It was found that LFA-1/CD18 and ICAM-1 play a significant role in the TMF/thymocyte adhesion. In addition, a LFA-1-dependent/ICAM-1-independent adhesion pathway was observed, suggesting that LFA-1 might use another ligand. The inhibitory effect of anti-CD18 mAb (WT-3) was higher than the effect of anti-LFA-1 mAb (WT-1) and was a consequence of blocking the CD18 chain both on thymocytes and TMF. No significant difference in the expression and function of adhesion molecules was found between TMF obtained from normal or hydrocortisone-treated rats. The involvement of CD11b in these processes was of lesser importance than the role of the CD11a molecule. By using mAbs to different epitopes of the CD11b molecule, such as OX-42 (anti-CD11b/CD11c), ED7, and ED8 (anti-CD11b), it was found that they were either slightly or moderately inhibitory under certain experimental conditions or did not significantly modulate TMF/thymocyte binding. OX-42 was slightly stimulatory in some experiments. Cumulatively, these results show that β2 integrins play a significant role in TMF/thymocyte interactions and probably contribute to T-cell development *in vivo*.

KEYWORDS: Thymic macrophages, thymocytes, adhesion molecules, cytokines, rosettes.

### INTRODUCTION

Thymic macrophages (TMF) and dendritic cells (DC) constitute a significant component of the thymic microenvironment (Kyewski, 1988). They are believed to originate from common precursors in the bone marrow, which, entering the thymus, probably transform into morphologically, phenotypically, and functionally different populations under the influence of different microenvironmental stimuli in the thymus (Duijvestijn et al., 1984; Kampinga and Aspinall, 1990). However, very recent data suggest that mouse thymic DC, unlike TMF, may differentiate from the $CD4^{low}$ thymic lymphoid precursor cells (Ardavin et al., 1993). In situ, TMF are located in the cortex, corticomedullary zone (CMZ), medulla, and connective tissue septa. Their morphology, expression of class II MHC molecules, and phagocytic function vary, depending on the topographical localization in the thymus (Zepp et al., 1984; Hamblin and Edgeworth, 1988). In the rat, cortical and CMZ macrophages clearly differ by their ultrastructure and enzyme histochemical properties (Milićević, 1984; Milićević et al., 1987). In addition, hibridoma technology enabled the production of mAbs that selectively recognize cortical and medullary macrophages (Dijkstra et al., 1985; Čolić et al., 1990).

*Corresponding author.

TABLE 1
Immunoreactivity of mAbs on the Rat Thymus[a]

| mAb | Isotype | Specificity | Staining pattern | References |
|---|---|---|---|---|
| ED1 | IgG1 | 97 kD protein | All TMF[c] | Dijkstra et al., 1985 |
| R-MC 41 | IgG1 | ND[b] | Cortical/CMZ[d]TMF[c] | Ćolić et al., 1990 |
| R-MC 43 | IgM | ND[b] | CMZ/medullary TMF[c] | Ćolić et al., 1990 |
| WT-1 | IgG2a | CD11a | Thymocytes, granulocytes | Tamatani et al., 1991a |
| WT-3 | IgG1 | CD18 | Thymocytes, TMF[c], DC, granulocytes | Tamatani et al., 1991a |
| 1A29 | IgG1 | CD54 | Thymic epithelium, TMF[c], DC, endothelium, subset of thymocytes | Tamatani and Miyasaka, 1990 |
| OX-42 | IgG2a | CD11b/c | Subset of TMF[c], granulocytes | Robinson et al., 1986 |
| ED7 | IgG1 | CD11b | Subset of TMF[c], granulocytes | Damoiseaux et al., 1989 |
| ED8 | IgG1 | CD11b | Subset of TMF[c], granulocytes | Damoiseaux et al., 1989 |
| IF119 | IgM | Certain MHC class II epitopes | DC[e], cortical epithelium (weakly) | Nagelkerken et al., 1987 |

[a] Staining patterns are given in original references and additionally checked in cryostat sections of adult AO rats at the Institute of Medical Research, MMA, Belgrade. [b] ND: not determined.[c] TMF-thymic macrophages, [d]CMZ-cortico-medullary zone, [e]DC-dendritic cells.

It was shown that macrophages form complexes with thymocytes in a rosette form (Epstein et al., 1985; Kyewski, 1988; Zeira and Gallily, 1988; Čolić and Drabek, 1991). It is supposed that these close cellular contacts influence the T-cell repertoire and selection processes in the thymus (Adkins et al., 1986; Kyewski, 1988). The contacts between thymic nonlymphoid cells and thymocytes are mediated by different adhesion molecules (Haynes, 1990). β2 integrins that belong to the integrin superfamily of adhesion molecules and are composed of the common β2 (CD18) chain and three distinct but very homologous α chains (αL-CD11a, αM-CD11b, and αX-CD11c) are of particular importance (Springer, 1990). Being widely distributed on various leukocytes, they participate in many intercellular adhesive interactions and have a capacity for signal transduction (Haynes, 1992). However, the adhesion molecules involved in the interaction between TMF and thymocytes are poorly understood. In this work, we present a significant role of β2 integrins in these processes using a short-term culture of almost pure population of rat TMF.

### RESULTS

### Phenotypic Characteristics of Rat TMF in Short-Term Culture

Adherent cells from short-term culture of thymic cell suspensions possessed typical macrophage morphology. This was confirmed using ED1 mAb (a pan-macrophage marker in the rat) (Table 1), which showed that 94 ± 2% of the cells isolated from normal thymus were ED1$^+$ (Table 2; Figs. 1a and 1b). The use of mAbs specific for particular TMF populations (R-MC 41 and R-MC 43) showed that the proportion of the TMF-bearing cortical phenotype was more numerous (38 ± 6%) than the relative values of R-MC 43$^+$ TMF originating from the medulla (13 ± 6%). DC, as determined using 1F119 mAb, were not detected. No significant difference in the phenotype of TMF obtained from normal and hydrocortisone-treated rats was found.

### Expression of β2 Integrins and ICAM-1 on TMF

We next studied the expression of β2 integrins and ICAM-1 on the cultivated rat TMF by immunocytochemistry and found (Table 3) that most of them isolated from normal thymus expressed CD11b, defined by ED7 mAb (83%), CD11b/CD11c defined by OX-42 mAb (93%), and CD18 molecules defined by WT-3 mAb (85%), whereas CD11a (WT-1 mAb) (Fig. 1c) was present on a small TMF population (20%). ICAM-1, as assessed by 1A29 mAb, was expressed on 69% TMF.

The number of LFA-1$^+$ (Fig. 1d), CD18$^+$, and ICAM-1$^+$ TMF increased after the 2-day stimulation of TMF with IFN-γ or TNF-α. The percentage of CD11b$^+$ TMF was not significantly changed, but

TABLE 2
Phenotypic Characteristics of Rat TMF in Short-Term Culture

| MAb | % positive cells[a] Normal thymus | Hydrocortisone-treated thymus |
|---|---|---|
| ED1 | 94 ± 2 | 96 ± 2 |
| R-MC 41 | 38 ± 6 | 36 ± 5 |
| R-MC 43 | 13 ± 6 | 11 ± 4 |

[a] The percentages of positively stained TMF are given on the basis of 200–300 calculated cells. They represent the mean of triplicate ± S.D. (normal thymus) or the mean ± S.D. from three independent experiments (hydrocortisone-treated thymus).

TABLE 3
Expression of β2 Integrins and ICAM-1 on Rat TMF in Short-Term Culture

| | % postive cells[a] | | | | | |
|---|---|---|---|---|---|---|
| | Normal thymus | | | Hydrocortisone-treated thymus | | |
| mAb | Medium | IFN-γ | TNF-α | Medium | IFN-γ | TNF-α |
| WT-1 | 18 ± 3 | 47 ± 8 | 29 ± 4 | 13 ± 6 | 51 ± 12 | 28 ± 5 |
| WT-3 | 84 ± 4 | 88 ± 5 | 92 ± 2 | 79 ± 5 | 88 ± 3 | 90 ± 3 |
| OX-42 | 89 ± 5 | 92 ± 2 | 92 ± 3 | 92 ± 4 | 92 ± 2 | 93 ± 2 |
| ED7 | 80 ± 5 | 85 ± 3 | 86 ± 5 | 72 ± 5 | 82 ± 3 | 81 ± 4 |
| ED8 | 79 ± 6 | 80 ± 5 | 82 ± 3 | 82 ± 3 | 87 ± 2 | 88 ± 3 |
| 1A29 | 68 ± 6 | 90 ± 3 | 85 ± 1 | 65 ± 4 | 89 ± 4 | 80 ± 4 |

[a]The percentages of positively stained TMF are given on the basis of 200–300 calculated cells. They represent the mean ± S.D. from three independent experiments.

the majority of cells were more strongly labeled with both ED7 and OX-42 mAbs (Figs. 1e and 1f). No significant difference in the expression of adhesion molecules between TMF cultivated from normal and hydrocortisone-treated rat thymuses was observed.

### Expression of β2 Integrins and ICAM-1 on Thymocytes

The expression of β2 integrins and ICAM-1 was also studied on thymocytes by flow cytometry. Figure 2 shows that the majority (92–97%) of resting rat thymocytes expressed both the LFA-1 and CD18 molecules. In contrast, only one subset of thymocytes (10–20%) was ICAM-1$^+$. The expression of the LFA-1 and CD18 molecules on Con A + IL-2–activated thymocytes did not significantly change, whereas about 75–80% activated thymocytes were ICAM-1$^+$. Both resting and activated thymocytes were CD11b (OX-42, ED7) negative (data not shown).

### Effect of IFN-γ and TNF-α on Rosette Formation between TMF and Thymocytes

The adhesion between TMF and thymocytes was studied using a rosette assay. Figure 3 shows that about 30–35% of unstimulated TMF bound to both resting and Con A + IL-2–activated thymocytes. In two separate experiments using TMF from hydrocortisone-treated rats, we showed that the percentage of R-MC 41$^+$ rosettes was slightly lower (31 ± 4%) than the percentage of TMF that did not bind thymocytes (42 ± 6%). The difference in the expression of R-MC 43 antigen was not significant (17 ± 3%) of TMF with rosetting thymocytes; 18 ± 4% free TMF). The binding of thymocytes (especially the activated ones) increased (3 hr) and was relatively constant thereafter (up to 5 hr) (data not shown).

The stimulation of TMF with cytokines (IFN-γ and TNF-α) incresed both early (30 min) and late (3 hr) adhesion of resting thymocytes (Fig. 3). The most pronounced effect was seen aftr 3 hr when TMF were stimulated with TNF-α (68 ± 8% rosettes) in comparison to the values of unstimulated TMF (42 ± 15% rosettes) ($p < 0.001$). The stimulation of TMF with these cytokines did not significantly modulate the binding of activated thymocytes ($p > 0.05$).

### Role of β2 Integrins and ICAM-1 in the Binding of Thymocytes to Unstimulated TMF

The effects of mAbs against the rat β2 integrins and ICAM-1 (all at the concentration of 10 μg/ml) on rosette formation between thymocytes and unstimulated TMF were given in Fig. 4.

When resting thymocytes were used, both WT-1 (anti-LFA-1) and WT-3 (anti-CD18) mAbs showed significant inhibitory effect (35–40% and 50–60% respectively), which was independent of incubation time. In contrast, 1A29 (anti-ICAM-1) mAb showed a slight inhibitory effect after 30 min (15–20%), which significantly increased after 3 hr of incubation. MAbs to CD11b were slightly stimulatory in some experiments (OX-42) or did not significantly change thymocyte binding to TMF (ED7 and ED8). No significance differences in the effects of mAbs on thymocyte binding to TMF from normal or hydrocortisone-treated animals were observed. The increase in mAb concentrations (30 μg/ml) did not potentiate the effect seen using 10 μg/ml of mAbs (data not shown).

We next preincubated WT-1 mAb separately with TMF and thymocytes and found that the inhibitory

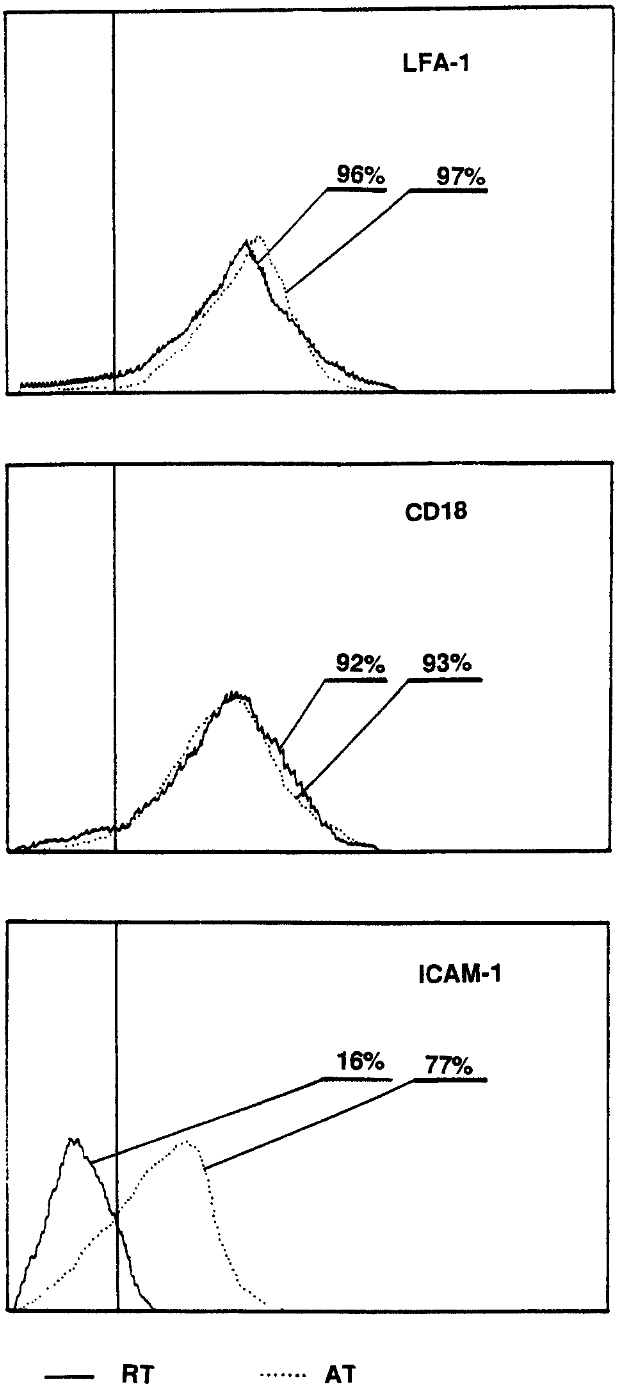

FIGURE 2 Flow cytometric analysis of resting (RT) and ConA + IL-2-activated (AT) thymocytes. Cells were labeled as described in Materials and Methods. Data shown are from one experiment representative of three experiments. Individual histograms were generated from $1 \times 10^4$ events. Vertical bars on histograms represent the level of background flourescence determined using an irrelevant mAb.

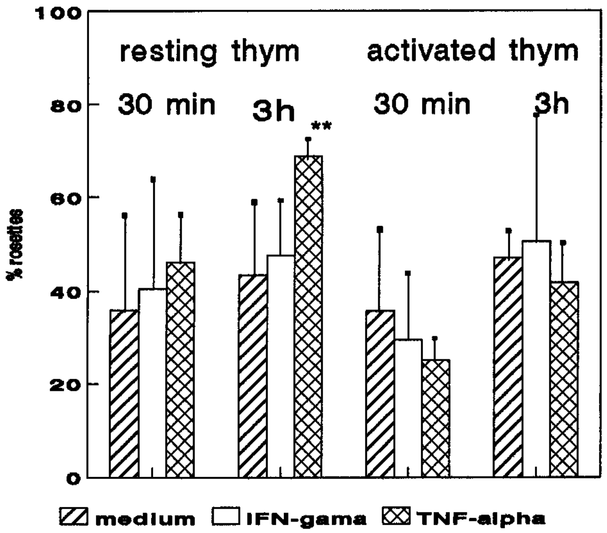

FIGURE 3 Effect of TMF stimulated with cytokines on thymocyte/TMF binding. Values (percentage of rosettes) are given as mean ± S.D. from three different experiments. TMF were stimulated for 2 days with 100 IU/ml of IFN-γ or 20 IU/ml of TNF-α. ** = $p < 0.05$ in comparison to percentage of rosettes formed between unstimulated TMF and resting thymocytes.

effect of this mAb was dependent on the LFA-1 expression on thymocytes (Fig. 4).

In order to determine whether the modulatory effect of mAbs on rosette formation was preferentially related to a distinct subset of TMF, we calculated the number of all thymocytes bound to TMF in control and in the samples with mAbs as well as the mean thymocyte number per rosette. The results presented in Table 4 clearly show that both the total number of bound thymocytes and the number of thymocytes per rosette were significantly lower using inhibitory mAbs (WT-1, WT-3, and 1A29) and were significantly higher using OX-42 mAb.

When Con A + IL-2-activated thymocytes were used, some differences were observed in comparison to the previous experiments. WT-3 was more inhibitory (especially after 3 hr of incubation) (75%). When thymocytes were preincubated with WT-3 mAb, the inhibition was 30 ± 12% after 30 min. Preincubation of TMF with this mAb resulted in 17 ± 6% inhibition, whereas 58 ± 14% inhibition was achieved when WT-3 was continuously present during the assay.

In contrast, 1A29 mAb was stimulatory (24%, 30 min; 50%, 3 hr). This effect was dependent on ICAM-1 expression on thymocytes but not on TMF. To exclude possible involvement of Fc receptors on TMF in this process, we preincubated TMF with

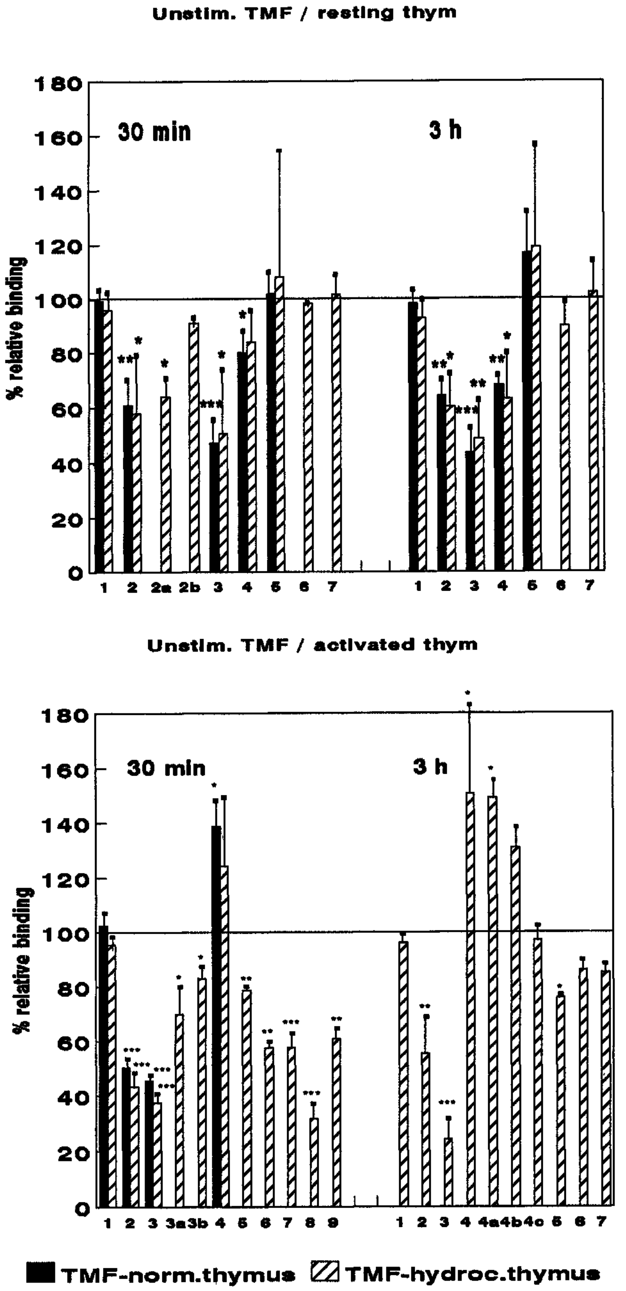

FIGURE 4 Effect of mAbs against β2 integrins and ICAM-1 on rosette formation between unstimulated TMF and thymocytes. Values (mean ± S.D. from three to four different experiment) are given as the percentage of relative binding to control (without mAb); * = $p < 0.05$; ** = $p < 0.005$; *** = $p < 0.001$ in comparison to an irrelevant mAb (IrmAb). 1.IrmAb; 2. WT-1; 2a. thymocytes preincubated with WT-1; 2b.TMF preincubated with WT-1; 3. WT-3; 3a. thymocytes preincubated with WT-3; 3b. TMF preincubated with WT-3; 4. 1A29; 4a. thymocytes preincubated with 1A29; 4b. TMF preinucbated with rat Ig and thymocytes preincubated with 1A29; 4c. TMF preincubated with 1A29; 5. OX-42; 6. ED7; 7. ED8; 8. WT-1 + ED7; and 9. OX-42 + ED7.

saturating amounts of rat Ig before the addition of thymocytes preincubated with 1A29 mAb. As shown in Fig. 4, this treatment decreased but not totally prevented the stimulatory effect of the mAb.

MAbs to CD11b were inhibitory. The inhibitory effect of ED7 and ED8 mAb was more visible and higher after 30 min of incubation (40%) than the effect of OX-42 mAb (20% inhibition). The combination of WT-1 and ED7 mAbs at this time potentiated the inhibition seen using single mAbs, whereas the combination of OX-42 and ED7 mAbs was without an additive inhibitory effect.

### Role of β2 Integrins and ICAM-1 in the Binding of Thymocytes to IFN-γ = stimulated TMF

When IFN-γ = stimulated TMF and resting thymocytes were incubated with mAbs, the inhibitory effects of WT-1 (50%) and WT-3 (60–65%) both after 30 min and 3 hr, and ICAM-1 after 30 min (30%) were higher (Fig. 5) than in the case when unstimulated TMF were used (Fig. 5). The inhibitory effects of anti-CD11b mAbs depended on mAbs used and incubation time. OX-42 mAb was moderately inhibitory (30%) after 30 min, whereas ED7 and ED8 inhibited thymocyte binding to TMF (30%) after 3 hr.

WT-1 and WT-3 also significantly inhibited the binding of activated thymocytes to IFN-γ stimulated TMF (40–70%). The effect was higher after 3 hr than after 30 min. The inhibitory effect of 1A29 was moderated (35%), but only after 30 min of cell incubation. OX-42, ED7, and ED8 did not significantly modulate thymocyte binding to TMF in these experiments even when these mAbs were applied at 50 μg/ml.

### Role of β2 Integrins and ICAM-1 in the Binding of Thymocytes to TNF-α-stimulated TMF

The initial binding (30 min) of resting thymocytes to TNF-α = stimulated TMF was significantly inhibited by WT-1 (50%), WT-3 (65%), and 1A29 mAb (55%) (Fig. 6). The effect of these mAbs significantly decreased after 3 hr (35%, 45%, and 20%, respectively). Of mAbs to CD11b, only OX-42 mAb was partially inhibitory after 30 min (25%), whereas other two mAbs (ED7 and ED8) were without significant effect.

TABLE 4
Effect of mAbs Against β2 Integrins and ICAM-1 on Thymocyte Binding to Unstimulated Rat TMF[a]

| | % rosettes | Number of adherent thymocytes/100 TMF | Number of adherent thymocytes/rosette[b] |
|---|---|---|---|
| Control | 33.7 | 208 | 6.4 |
| WT-1 | 19.9 (40.8%↓) | 94 (54.9%↓) | 3.8 |
| WT-3 | 11.8 (64.8%↓) | 81 (59.8%↓) | NT[c] |
| 1A29 | 15.6 (53.7%↓) | 110 (47.1%↓) | 3.9 |
| OX-42 | 59.2 (75.5%↑) | 399 (92.7%↑) | 8.7 |
| ED7 | 29.9 (11.1%↓) | 222 (7.6%↑) | 6.9 |
| ED8 | 30.3 (10.1%↓) | 205 (1.2%↓) | NT[c] |

[a]TMF were isolated from hydrocortisone-treated rats. Resting thymocytes were incubated with TMF for 3 hr as described. All parameters were calculated on the basis of 300 TMF. Values (mean of duplicate) are from one representative experiment. Numbers in parenthesis represent percentage of inhibition(↓) or stimulation (↑) compared to control. [b]The number of adherent thymocytes was calculated only in TMF-forming rosettes (3 and more thymocytes bound to a TMF).[c]NT = not tested.

Inhibitory effects of WT-1 and WT-3 mAbs were also strong when activated thymocytes were used (55–60%; 70–75%) and were independent of incubation time. In contrast, 1A29 and OX-42 mAbs were moderately inhibitory (30%) only after 3 hr of incubation.

### DISCUSSION

In this paper, we present a role of β2 integrins and one of their ligands, ICAM-1, on thymocyte binding to the rat TMF.

TMF were isolated as adherent cells from short-term cultures of thymic cell suspensions. This very simple method enabled the isolation of almost pure TMF that were 94–98% positive with ED1 mAb. This mAb is a pan-macrophage marker in the rat (Dijkstra et al., 1985). Among them, no dendritic cells, as determined by the use of 1F119 mAb, were identified (Nagelkerken et al., 1987). In addition, the use of thymic cell suspensions from *in vivo* hydrocortisone-treated rats, which were significantly depleted of thymocytes and enriched in TMF, enabled direct growth of TMF on microscopic slides. This procedure also simplified the rosette assay used for studying adhesion characteristics of TMF. Although hydrocortisone can induce changes in the expression of some adhesion molecules (Rothlein et al., 1988; Čolić and Drabek, 1993), we found that under experimental conditions described here, this hormone did not significantly alter the phenotype and adhesion molecules of TMF.

The short-term culture of adherent TMF has also some advantages because it could better reflect TMF characteristics *in vivo*. The results presented in our previous papers (Čolić et al., 1991; Čolić and Drabek, 1991) demonstrated that longer cultivation of TMF (2–3 weeks) using an explant technique or serum-free medium enabled significant proliferation of TMF that differed by some phenotypic and adhesion characteristics from the adherent TMF used in this study.

We demonstrated that the majority of adherent TMF expressed the CD11b molecule defined by OX-42, ED7, and ED8 mAbs, and the CD18 molecule defined by WT-3 mAb. OX-42 mAb also recognized an epitope expressed on the rat CD11c molecule (Tamatani et al., 1991a). The expression of CD11b on cultivated TMF was higher than its expression *in situ* (Robinson et al., 1986; Damoiseaux et al., 1989; our unpublished observations) or on freshly isolated TMF (50–56%) (Damoiseaux et al., 1989), suggesting that the upregulation of CD11b expression could be induced in culture. We also found that most (60–70%) TMF expressed relatively low levels of ICAM-1. A similar percentage of the ICAM-1$^+$ freshly isolated rat TMF was previously demonstrated (Čolić and Drabek, 1991). A small population of adherent TMF expressed a detectable amount of the LFA-1 molecule, which has not been reported for TMF so far.

Expression of all these molecules was upregulated by the influence of IFN-γ and TNF-α. These inflammatory cytokines are potent inducers of the ICAM-1 expression on many nonlymphoid cell lines (Rothlein et al., 1988). TNF-α upregulated the expression of CD11b on granulocytes and monocytes (Griffin et al., 1990). However, their modulatory effects on TMF adhesion molecules have not been extensively studied before. We hypothesized that the use of TMF stimulated with IFN-γ and TNF-α is of importance to understand TMF/thymocyte interactions *in vivo*, because these cytokines are locally produced in the thymus (Ritter and Boyd, 1993).

In this study, we demonstrated that β2 integrins and ICAM-1 play a significant role in TMF/thymocyte interactions. Among them, the LFA-1

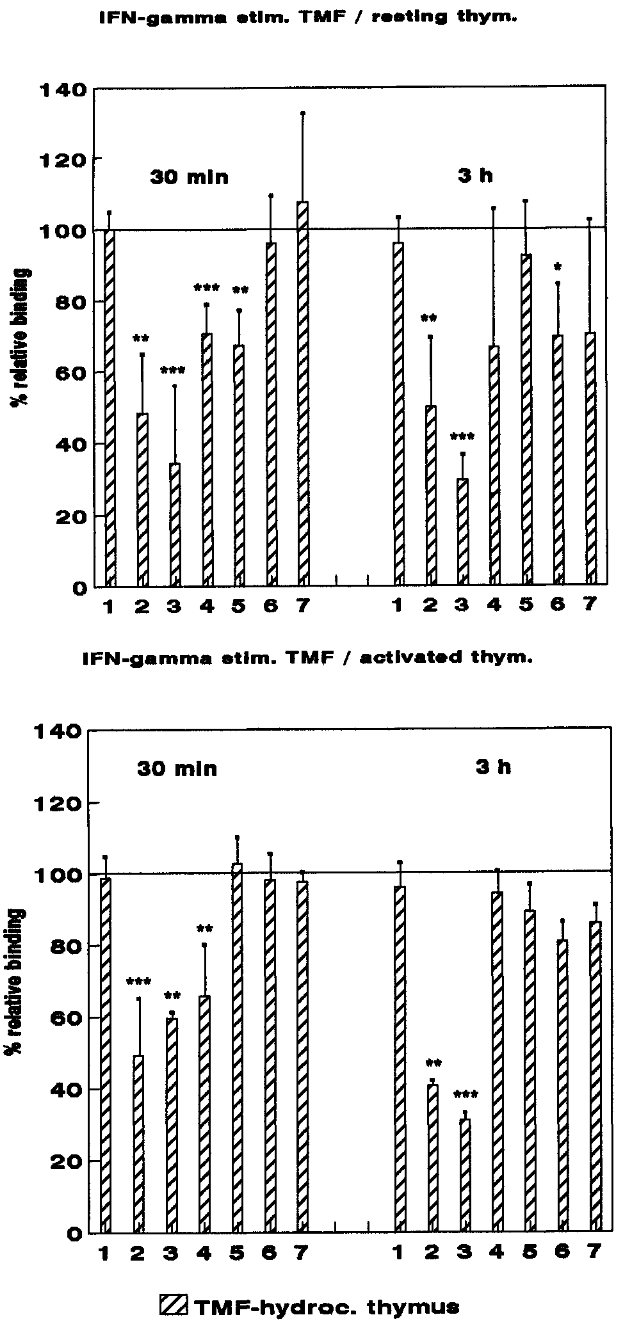

FIGURE 5 The effect of mAbs against β2 integrins and ICAM-1 on rosette formation between IFN-γ-stimulated TMF and thymocytes. TMF were isolated from hydrocortisone-treated rats. 1. IrmAb; 2. WT-1; 3. WT-3; 4. 1A29; 5. OX-42; 6. ED7; and 7. ED8. The results are presented as in Fig. 4.

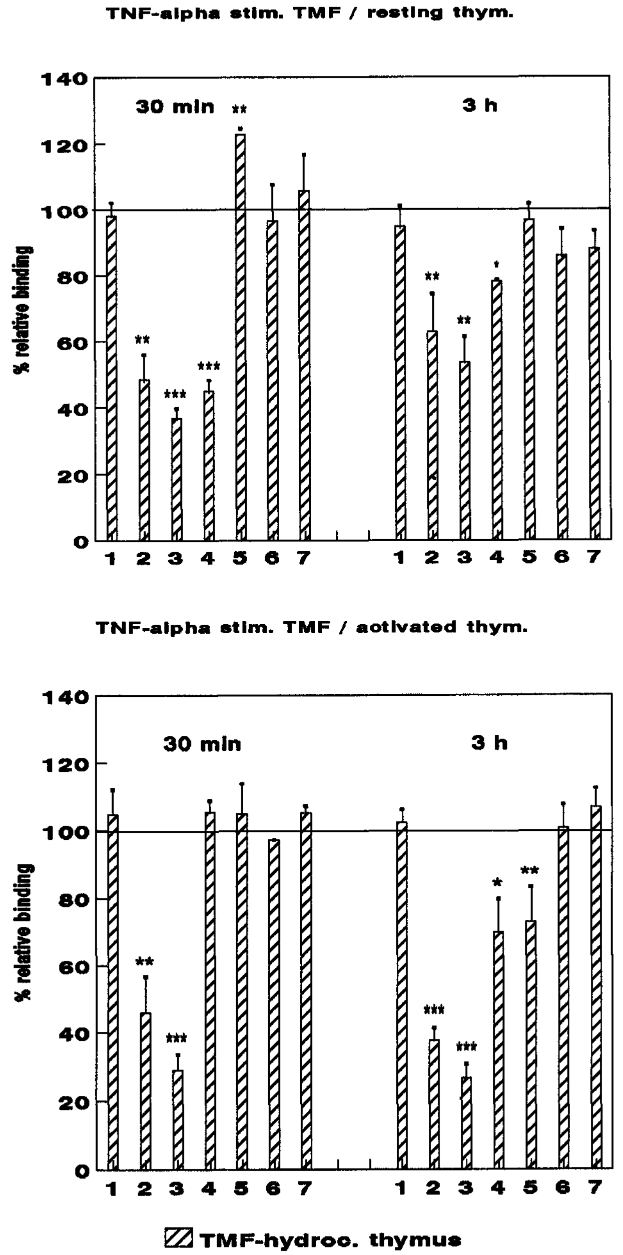

FIGURE 6 The effect of mAbs against β2 integrins and ICAM-1 on rosette formation between TNF-α-stimulated TMF and thymocytes. TMF were isolated from hydrocortisone-treated rats. 1. IrmAb; 2. WT-1; 3. WT-3; 4. 1A29; 5. OX-42; 6. ED7; and 7. ED8. The results are presented as in Fig. 5.

and CD18 molecules have a more important role than the CD11b molecule. Effects of mAbs directed to these antigens depended on their epitope specificity, incubation time, and activation state both of thymocytes and TMF.

The role of the LFA-1/ICAM-1 receptor-ligand system in thymocyte binding to thymic epithelial cells (TEC) has been well documented. Lepesant et al. (1990) showed a role of the LFA-1 molecule in the stabilization of a rapid adhesion phase between murine thymocytes and a TEC (MTE) line. Other authors reported different results showing the significance of the LFA-1 molecule in the binding of resting rat thymocytes to an unstimulated TEC line (Kinebuchi et al., 1991), the absence of inhibition by anti-LFA-1 mAbs when cultured TEC did not express ICAM-1 (Nonoyama et al., 1989), and the involvement of LFA-1 in the adhesion of Con A + IL-2–activated human thymocytes, but not resting thymocytes, to IFN-γ-stimulated TEC (Singer et al., 1990).

The involvement of the LFA-1/ICAM-1 receptor/ligand system in TMF/thymocyte interactions has not been extensively studied. The only previously published work on the involvement of the LFA-1/ICAM-1 receptor-ligand system in TMF/thymocyte interactions (Ćolić and Drabek, 1991) showed a significant LFA-1-dependent/ICAM-1-dependent adhesion pathway between thymocytes and rat TMF cultivated for 2 weeks in serum-free medium and stimulated with IFN-γ. In this work, experiments were extended using unstimulated and stimulated TMF as well as resting and activated thymocytes. We obtained very interesting results that clearly showed the involvement of a LFA-1-dependent/ICAM-dependent and LFA-1-dependent/ICAM-1-independent adhesion pathways. Their involvement depended on incubation time and activation state, both of thymocytes and TMF. Similar results have not been published for the interactions between thymocytes and thymic nonlymphoid cells but were demonstrated using the same WT-1 and 1A29 mAbs in the binding between rat T lymphocytes and a high endothelial venule (HEV) cell line (Tamatani et al., 1991b).

The typical LFA-1/ICAM-1-dependent adhesion was observed using TNF-α-stimulated TMF and resting thymocytes (30 min). The typical LFA-1-dependent/ICAM-1-independent adhesion was seen using unstimulated TMF and activated thymocytes. Other TMF/thymocyte combinations probably involved both of these adhesion pathways. These results indicate that LFA-1 could use other ligands such as ICAM-2 (Staunton et al., 1989) or ICAM-3 (Fawcett et al., 1992). However, their expression on thymic nonlymphoid cells is not sufficiently known and mAbs to these molcules in the rat are not available yet.

In these experiments, we also demonstrated that the IFA-1/ICAM-1 molecules are not significant only in the first adhesion phase as reported for thymocyte/TEC interactions (Lepesant et al., 1990), but also in the late adhesion phase (3 hr of incubation). A similar role of the LFA-1 antigen in the binding between human dendritic cells and lymphocytes was demonstrated after 4 hr of cell incubation (Scheeren et al., 1991).

An interesting phenomenon presented in this work concerns the stimulatory effect of anti-ICAM-1 (1A29) mAb on the rosette formation between TMF and activated thymocytes. We demonstrated that this effect depended on the ICAM-1 expression on activated thymocytes and was only partly mediated by the interaction between Fc receptors on TMF and 1A29 mAb coated onto thymocytes. One possible explanation is that 1A29 mAb could generate signals (Gronberg et al., 1993) that induce conformational changes of the ICAM-1 or LFA-1 molecules. Such a change in the avidity of LFA-1 is well documented by stimulation of lymphocytes with anti-CD3 and anti-CD2 mAbs (van Kooyk et al., 1989) as well as by mAbs to some epitopes of the LFA-1 molecule (Keizer et al., 1988). The next experiments will be designed to answer this question.

The difference in the binding between resting and activated thymocytes to TMF could be a consequence of the higher expression of ICAM-1 on activated thymocytes (Fig. 3) and probably of an increased avidity of LFA-1 for its ligands that was demonstrated on spleen cell Con A blasts (Tamatani et al., 1991a). Another possibility may represent the distinct subset composition found in resting and activated thymocytes. In our previous work (Čolić and Drabek, 1991), we demonstrated that resting thymocytes attached to TMF are enriched in immature double negative ($CD4^{-}CD8^{-}$) and double positive ($CD4^{+}CD8^{+}$) cells. However, among activated thymocytes, a significant proportion of mature single positive ($CD4^{+}$ or $CD8^{+}$) cells was present (Čolić et al., in preparation).

El Rouby et al. (1985) described that the binding between mouse thymocytes and phagocytic cells of the thymic reticulum (P-TR) is almost completely inhibited using Mac-1 (anti-CD11b) mAb. In our experiments, it was demonstrated that CD11b is of lesser importance in TMF/thymocyte binding than the CD11a molecule. The difference could be explained by the fact that P-RT and adherent TMF do not represent the same population, that Mac-1 and our mAbs used (OX-42, ED7 and ED8) do not recognize the same epitopes on corresponding molecules, or the affinity of the mAbs applied is not the same.

We also found that the effect of anti-CD11b mAbs depended on their epitope specificity, incubation time of cells, and activation of thymocytes or TMF. ED7 and ED8 mAbs were slightly or moderately inhibitory under certain conditions or did not significantly modulate TMF/thymocyte binding. OX-42 mAb had a similar effect, but in some experiments, it was stimulatory. The reason for these differences is not clear. It can be hypothesized that the adhesion between TMF and thymocytes is a dynamical process in which the epitopes both of CD11b and other adhesion molecules studied are differentially involved.

In conclusion, this work shows that β2 integrins and ICAM-1 play a significant role in TMF/thymocyte interactions and probably contribute to T-cell development *in vivo*. However, the combination of mAbs to these molecules did not completely block thymocyte binding to TMF, suggesting the involvement of some other adhesion molecules.

### MATERIALS AND METHODS

### Animals

AO rats, both sexes, 8–10 weeks old, bred at the Farm for Experimental Animals, Military Medical Academy, Belgrade, were used in this study.

### In Vivo Hydrocortisone Treatment

Hydrocortisone acetate (ICN-Galenika, Belgrade) (150 mg/kg) was injected i.p. into rats 2 days before their sacrifice.

### Thymocyte Preparation

Thymuses from normal or hydrocortisone-treated rats were teased against a steel mesh. Released cells were collected and resuspended in RPMI-1640 medium (Serva, Germany) containing 10% fetal calf serum (FCS) (Flow) and 1% garamicin (ICN Galenika, Belgrade) to obtain a single cell suspension. The suspension was filtered through a nylon gauze to remove fibrous residues. Cell viability was determined by trypan blue dye exclusion and was more than 95%. Activated thymocytes were prepared by the 3-day cultivation of thymocytes with Con A (1 μg/ml) and recombinant IL-2 (10 IU/ml).

### Short-Term Cultivation of TMF

Cells from normal thymuses ($5 \times 10^6$/ml) were cultivated for 3 days in 25 $cm^2$ plastic flasks (Flow) in 10 ml of medium. Nonadherent cells were discarded and adherent cells, which usually represent 0.03–0.08% of total thymic cells, were detached from the plastic surface using cold 0.04% EDTA in PBS and vigorous pipetting. After washing in RPMI-1640 medium, adherent cells were counted, resuspended in 50 μl of complete medium, placed onto 8-mm-diameter sterile multispot glass slides (Flow) ($1–2 \times 10^3$ cells/spot), placed in petri dishes, and cultivated in an incubator with 5% $CO_2$ at 37°C for 24 hr.

Thymic cells from hydrocortisone-treated rats ($5 \times 10^5$) were resuspended in 50 μl of complete medium, pipetted directly onto 8-mm-diameter slides, and cultivated as described earlier. The medium was changed after 2 days. After the additional 2-day cultivation, slides with adherent cells, which usually represent 0.2–0.4% of total thymic cells, were gently washed in PBS and either air dried and further processed for immunocytochemistry or used for a rosette assay as described.

In experiments using stimulated TMF, adherent cells on glass slides were incubated for 2 days with recombinant IFN-γ (100 IU/ml) or TNF-α (20 IU/ml). The concentration of cytokines was determined on the basis of their known activities given by suppliers.

### Antibodies and Cytokines

A panel of mAbs was used. Their specificities, isotypes, and staining patterns on the rat thymus are given in Table 1. R-MC 41 and R-MC 43 mAbs were produced at the Institute of Medical Research, MMA, Belgrade (Čolić et al., 1990). They are specific for cortical/CMZ and CMZ/medullary TMF, respectively. The ED series of mAbs, such as ED1 (as panmacrophage marker) (Dijkstra et al., 1985), ED7, and ED8 (reactive with rat CD11b) (Damoiseaux et al., 1989), was a kind gift from Dr. C. Dijkstra. ED7 partially inhibited T-lymphocyte clustering with rat dendritic cells, whereas ED8 mAb did not show any inhibitory effect in this assay (Damoiseaux, 1991). WT-1 (anti-rat LFA-1), WT-3 (anti-rat CD18) (Tamatani et al., 1991a), and 1A29 mAb (anti-rat ICAM-1) (Tamatani and Myasaka, 1990) were produced in Tokyo. All these mAbs recognize "inhibitory" epitopes on the corresponding molecules (Tamatani et al., 1991a; Tamatani and Miyasaka, 1990). OX-42 mAb (anti-rat CD11b/c) (Robinson et al., 1986; Tamatani et al., 1991a) was obtained from Serotec, and the 1F119 mAb (Nagelkerken et al., 1987) reactive with rat DC was a generous gift from Dr. L. Nagelkerken. Secondary biotinylated antibody (goat anti-mouse Ig) and streptavidin-peroxidase were purchased from Amersham International (Amersham, UK). Sheep anti-mouse Ig-FITC was obtained from INEP (Zemun, Yugoslavia). Recombinant rat IFN-γ was a generous gift from Dr. P. van der Meide (TNO, Rijswik, The Netherlands). Human recombinant IL-2 and mouse recombinant TNF-α were obtained from Genzyme (Boston).

### Immunocytochemistry

Monolayers of adherent cells were fixed in acetone and incubated with appropriate dilutions of mAbs for 60 min, followed by blocking of endogeneous peroxidase activity in methanol containing 0.3% $H_2O_2$. After that, monolayers were incubated with sheep anti-mouse Ig biotinylated antibody (1:100 in TBS) with addition of 5% normal rat serum for 30 min and then with streptavidin peroxidase (1:100 in TBS) for 30 min. After washing with TBS, the slides were stained for peroxidase activity with 0.06% DAB and 0.01% $H_2O_2$. Finally, monolayers were counterstained with hematoxylin and observed under a light microscope (Jenamed). Control monolayers were incubated in the same way, using mouse isotype-matched irrelevant mAbs (produced in MMA, Belgrade).

### Flow Cytometry

Thymocytes (1 x $10^6$) were stained in suspension with mAbs and secondary anti-mouse Ig-FITC using a classical indirect immunofluorescence assay. An irrelevant mouse Ig was used as control. The cells were then analyzed on an EPICS-CS flow cytometer (Coulter, Germany).

### Rosette Assay for TMF-Thymocyte Binding

Thymic cells from normal or hydrocortisone-treated rats cultivated on multispot glass slides were washed in PBS to remove nonadherent cells. Adherent cells (TMF) were incubated with 5 x $10^5$ resting thymocytes or 2 x $10^5$ activated thymocytes for 30 min or 3 hr at 37°C. A smaller number of activated thymocytes was used for their greater size in comparison to the resting cells. After that, slides were fixed in glutar-aldehyde (1% in PBS) for 20 min and then washed for 30 min in PBS. During this time, nonbound thymocytes were removed. After that, slides were stained with hematoxylin-eosin and analyzed under a light microscope. TMF that bound 3 or more thymocytes were scored as rosettes. For each assay, 300–500 macrophages were counted and each determination was performed in triplicate. The results are given as mean of rosette percentages.

For blocking experiments, TMF were preincubated with 1A29, OX-42, ED7, and ED8 mAbs, whereas thymocytes were preincubated with WT-1 and WT-3 mAbs at 4°C for 30 min. Both TMF and thymocytes were preincubated with an irrelevant mAb. After the addition of thymocytes to the adherent TMF, mAbs were present throughout the assay. In some experiments, WT-1, WT-3, and 1A29 mAbs were separately preincubated with TMF or thymocytes and then an excess of mAbs was removed by washing before the rosette assay.

The results are presented as

$$\% \text{ relative binding} = \frac{\text{number of rosettes with mAb}}{\text{number of rosettes without mAb}} \times 100$$

For statistical analysis (Student *t*-test), the percentage of relative binding in the presence of specific mAb was compared with that using an irrelevant mAb.

In certain experiments, the number of thymocytes bound to TMF was calculated and presented as the number of adherent thymocytes/100 TMF. In addition, the mean number of thymocytes per rosette was also determined.

### ACKNOWLEDGMENTS

We thank Dr. P. van der Meide for providing us with recombinant rat IFN-γ, Dr. C. Dijkstra for ED1, ED7 and ED8 mAbs, Dr. L. Nagelkerken for 1F119 mAb and Dragan Babic and Marinko Djordjević for technical assistance.

*(Received July 16, 1993)*

*(Accepted December 12, 1993)*

### REFERENCES

Adkins B., Mueller C., Okada C., Reichert R.A., Weissman I.L., and Spahgrude G.J. (1986). Early events in T-cell maturation. Annu. Rev. Immunol. **5**: 325–365.

Ardavin C., Wu L., Li C-L., and Shortman K. (1993). Thymic dendritic cells and T cells develop simultaneously in the thymus from a common precursor population. Nature **362**: 761–763.

Čolić M., and Drabek D. (1991). Expression and function of intercellular adhesion molecule 1 (ICAM-1) on rat thymic macrophage in culture. Immunol. Lett. **28**: 251–258.

Čolić M., and Drabek D. (1993). Phenotype and adhesion characteristics of rat thymic macrophages cultivated in serum free medium. Thymus **21**: 43–60.

Čolić M., Popović Lj., Gašić S., Drabek D., and Dujić A. (1991). Primary culture of rat thymic non-lymphoid cells: Influence of culture time on expression of macrophage differentiation antigens defined by monoclonal antibodies. Thymus **18**: 243–256.

Čolić M., Popović Lj., Gašić S., Dragojević-Simić V., Milićević N.M., Matanović D., and Dujić A. (1990). Immunohistochemical characterization of rat thymic non-lymphoid cell. II. Macrophages and granulocytes defined by monoclonal antibodies. Immunology **69**: 416–422.

Damoiseaux J.G.M.C., Dopp E.A., Neefjes J.J., Beelen R.H.J., and Dijkstra, C.D. (1989). Heterogeneity of macrophages in rat evidenced by variability in determinants: Two new anti-rat macrophage antibodies against a heterodimer of 160 and 95 kd (CD11/CD18). J. Leukocyte Biol. **46**: 556–564.

Damoiseaux J.G.M.C. (1991). Macrophage heterogeneity in the rat. Doctoral thesis, Free University, Amsterdam, pp. 107–121.

Dijkstra C.D., Dopp E.A., Joling P., and Kraal G. (1985). The heterogeneity of mononuclear phagocytes in lymphoid organs: Distinct macrophage population in the rat recognized by monoclonal antibodies. Immunology **54**: 589–599.

Duijvestijn A.M., Sminia T., Kohler Y.G., Janse E.M., and Hoefsmit E.C.M. (1984). Ontogeny of the rat thymus microenvironment: Development of the interdigitating cells and macrophage populations. Dev. Comp. Immunol. **8**: 451–460.

El Rouby S., Praz F., Halbwacks-Mecarelli L., and Papiernik M. (1985). Thymic reticulum in mice: IV. The rosette formation between phagocytic cells of the thymic reticulum and cortical type thymocytes is mediated by complement receptor type III. J. Immunol. **134**: 3625–3631.

Epstein H.D., Mitchel D.S., Hunt J.S., and Wood G.W. (1985). Ia-positive macrophages bind and internalize viable lymphocytes in murine thymus. Cell Immunol. **95**: 15–34.

Fawcett J., Holness C.L., Needham L.A., Turley H., Gatter K.C., Mason D.Y., and Simmons D.L. (1992). Molecular cloning of ICAM-3, a third ligand for LFA-1, constitutively expressed on resting lymphocytes. Nature **360**: 481–484.

Griffin J.D., Spertini O., Ernst T.Y., Belvin M.P., Levin H.B., Kanakura Y., and Tedder T.F. (1990). Granulocyte-macrophage colony-stimulating factor and other cytokines regulate surface expression of the leukocyte adhesion molecule-1 on human neutrophils, monocytes and their precursors. J. Immunol. **145**: 576–584.

Gronberg A., Halapi E., Ferm M., Petersson M., and Patarroyo M. (1993). Regulation of lymphocyte aggregation and proliferation through adhesion molecule CD54 (ICAM-1). Cell. Immunol. **147**: 12–24.

Hamblin A.S., and Edgeworth J.D. (1988). Does antigen presentation occur in the thymus. *In*: Thymus update 1. The microenvironment of the human thymus, Kendall M.D. and Ritter, M.A., Eds. (London: Harwood Academic Publishers) pp. 135–153.

Haynes F.B. (1990). Human thymic epithelium and T-cell development: Current issues and future directions. Thymus **16**: 143–158.

Haynes R.O. (1992). Integrins: Versatility, modulation, and signaling in cell adhesion. Cell **69**: 11–25.

Kampinga J., and Aspinall R. (1990). Thymocyte differentiation and thymic micro-environment development in the fetal rat thymus: An immunohistological approach. *In*: Thymus update 3. The role of the thymus in tolerance induction, Kendall M.D. and Ritter M.A., Eds. (London: Harwood Academic Publishers) pp. 149–186.

Keizer G.D., Visser W., Vliem M., and Figdor C.G. (1988). A monoclonal antibody (NKI-L16) directed against a unique epitope on the α-chain of human leucocyte function-associated antigen 1 induces homotypic cell–cell interactions. J. Immunol. **140**: 1393–1400.

Kinebuchi M., Ide T., Lupin D., Tamatani T., Miyasaka M., Matsuura A., Nagai Y., Kikuchy K., and Uede T. (1991). A novel cell surface antigen involved in thymocyte and thymic epithelial cell adhesion. J. Immunol. **146**: 3721–3728.

Kyewski B., (1988). Unravelling the complexity of intrathymic cell-cell interaction. APMIS **96**: 1049–1060.

Lepesant H., Reggio H., Pierres M., and Naquet P. (1990). Mouse thymic epithelial cell lines interact with and select $CD3^{low}$ $CD4^+$ $CD8^+$ thymocyte subset trough an LFA-1 dependent adhesion-de-adhesion mechanism . Int. Immunol. **2**: 1021–1032.

Milićević N.M., and Milićević Ž. (1984). Enzyme histochemical characterization of macrophages in the rat thymus, with special reference to metallophilic cells of the cortico-medullary zone. J. Leukocyte Biol. **36**: 761–769.

Milićević N.M., Milićević Ž., Čolić M., and Mujović S. (1987). Ultrastructural study of macrophages in the rat thymus, with special reference to cortico-medullary zone. J. Anat. **150**: 89–98.

Nagelkerken L.M., Schutte B., Stet R.J.M., and Breda-Vriesman P.J.C. (1987). Recognition of rat dendritic cells by monoclonal antibody. Scand. J. Immunol. **27**: 347–371.

Nonoyama S., Nakayama M., Shijohara T., and Yata J.I. (1989). Only dull $CD3^+$ thymocytes bind to the thymic epithelial cells. The binding is eliciated by both CD2/LFA-3 and LFA-1/ICAM-1 interactions. Eur. J. Immunol. **19**: 1631–1635.

Ritter M.A., and Boyd R.L. (1993). Development in the thymus: It takes two to tango. Immunol. Today **14**: 462–469.

Robinson A.P., White T.M. and Mason D.W. (1986). Macrophage heterogeneity in the rat as delineated by two monoclonal antibodies MRC-OX-41 and MRC-OX-42, the latter recognizing complement receptor type 3. Immunology **57**: 239–247.

Rothlein R., Czajkowski M., O'Neil L.M., Marlin S.D., Mainolfi E., and Merluzz V.J. (1988). Induction of intercellular adhesion molecule 1 on primary and continuous cell line by proinflammatory cytokines. J. Immunol. **141**: 1665–1669.

Scheeren R.A., Koopman G., van der Baan S., Meijer C.J.L.M., and Pals S.T. (1991). Adhesion receptors involved in clustering of blood dendritic cells and T lymphocytes. Eur. J. Immunol. **21**: 1101–1105.

Singer K.H., Denning S.M., Wichard L.P., and Haynes, B.F. (1990). Thymocyte LFA-1 and thymic epithelial cell ICAM-1 molecules mediate binding of activated human thymocytes to thymic epithelial cells. J. Immunol. **144**: 2931–2939.

Springer T.A. (1990). Adhesion receptors of the immune system. Nature **346**: 425–433.

Staunton D.E., Dustin M.L., and Springer T.A. (1989). Functional cloning of ICAM-2, cell adhesion ligand for LFA-1 homologous to ICAM-1. Nature **339**: 61–64.

Tamatani T., and Miyasaka M. (1990). Identification of monoclonal antibodies reactive with the rat homologue of ICAM-1, and evidence for a differential involvement of ICAM-1 in the adherence of resting versus activated lymphocytes to high endothelial cells. Int. Immunol. **2**: 165–171.

Tamatani T., Kotani M., and Miyasaka M. (1991a). Characterization of rat leukocyte integrin, CD11/CD18, by the use of LFA-1 subunit-specific monoclonal antibodies. Eur. J. Immunol. **21**: 627–633.

Tamatani T., Kotani M., Tanaka T., and Miyasaka M. (1991b). Molecular mechanisms underlying lymphocyte recirculation. II. Differential regulation of LFA-1 in the interaction between lymphocytes and high endothelial cells. Eur. J. Immuno. **21**: 855–858.

Van Ewijk W. (1991). T cell differentiation is influenced by thymic microenvironments. Annu. Rev. Immunol. **9**: 591–615.

Van Kooyk Y., Van de Wiel-van Kemende P., Weder P., Kuijpers T.W. and Figdor C.D. (1989). Enhancement of LFA-1 mediated cell adhesion by triggering through CD2 and CD3 on T lymphocytes. Nature **324**: 811–813.

Zeira M., and Gallily R. (1988). Interaction between thymocytes and thymus-derived macrophages. Surface components participating in mutual recognition. Cell Immunol. **117**: 264–276.

Zepp F., Schilte-Wisserman H., and Mannhardt W. (1984). Macrophage subpopulations regulate intrathymic T-cell development. I: Ia positive macrophages augment thymocytes proliferation. Thymus **6**: 279-293.

